# Coronary Blood Flow Is Increased in RV Hypertrophy, but the Shape of Normalized Waves Is Preserved Throughout the Arterial Tree

**DOI:** 10.3389/fphys.2018.00675

**Published:** 2018-05-31

**Authors:** Yunlong Huo, Ghassan S. Kassab

**Affiliations:** ^1^PKU-HKUST Shenzhen-Hongkong Institution, Shenzhen, China; ^2^Department of Mechanics and Engineering Science, College of Engineering, Peking University, Beijing, China; ^3^California Medical Innovations Institute, San Diego, CA, United States

**Keywords:** pulsatile flow, right ventricular hypertrophy, right coronary arterial tree, Womersley-type model, Pulsatile wall shear stress

## Abstract

A pulsatile hemodynamic analysis was carried out in the right coronary arterial (RCA) tree of control and RV hypertrophy (RVH) hearts. The shape of flow and wall shear stress (WSS) waves was hypothesized to be maintained throughout the RCA tree in RVH (i.e., similar patterns of normalized flow and WSS waves in vessels of various sizes). Consequently, we reconstructed the entire RCA tree down to the first capillary bifurcation of control and RVH hearts based on measured morphometric data. A Womersley-type model was used to compute the flow and WSS waves in the tree. The hemodynamic parameters obtained from experimental measurements were incorporated into the numerical model. Given an increased number of arterioles, the mean and amplitude of flow waves at the inlet of RCA tree in RVH was found to be two times larger than that in control, but no significant differences (*p* > 0.05) were found in precapillary arterioles. The increase of stiffness in RCA of RVH preserved the shape of normalized flow and WSS waves, but increased the PWV in coronary arteries and reduced the phase angle difference for the waves between the most proximal RCA and the most distal precapillary arteriole. The study is important for understanding pulsatile coronary blood flow in ventricular hypertrophy.

## Introduction

There is compensatory vascular remodeling that accompanies RV hypertrophy (RVH) (Cooper et al., 1981; Manohar et al., 1981; Botham et al., 1984; Manohar, 1985; White et al., 1992; Kassab et al., 1993). In porcine model, systemic blood pressure in right coronary artery (RCA) was unchanged, but an increase of blood flow occurred in large epicardial branches at 5 weeks after pulmonary hypertension (Lu et al., 2011). Based on the measured morphometric data in arrested, vasodilated porcine heart of RVH, we carried out a steady-state flow analysis of arterial tree down to first capillary segments (Huo et al., 2007). The increase of blood flow was found to be caused by the compensatory growth of small vessels, which resulted in restoration of blood flow and wall shear stress (WSS) to normal level in the perfusion arterioles (diameter <100 μm) of RCA tree in the diastolic state of RVH hearts (Huo et al., 2007). The pulsatility of coronary blood flow is a significant hemodynamic feature (Fung, 1997; Nichols and McDonald, 2011). However, the pulsatile pressure-flow relationship has not been investigated in the coronary circulation during the progression of RVH. We have simulated the pulsatile blood flow in diastole in the absence of vessel tone in the entire coronary arterial tree of normal porcine hearts using the Womersley-type mathematical model (Huo and Kassab, 2006). Hence, the objective of present study is to carry out a complete pulsatile flow analysis in the coronary arterial tree of control and RVH hearts and to determine the effect of RVH on the flow and WSS waves in diastole in the absence of vessel tone.

Here, we hypothesized that the flow and WSS waves was preserved in RVH (i.e., similar patterns of normalized flow and WSS waves in vessels of various sizes). To test the hypothesis, pulsatile blood flows were computed by a Womersley-type numerical model (Huo and Kassab, 2006) in each vessel of the entire RCA tree down to the first capillary bifurcation (excluding the sub-tree distal to the posterior descending artery) of control and RVH hearts in diastole, which was reconstructed from morphometric data (i.e., vessel diameters, lengths and numbers) (Kassab et al., 1993). The experimental measurements of coronary wall thickness (Guo and Kassab, 2004) and stiffness (Garcia and Kassab, 2009) were also incorporated into the numerical model. The constitutive equation, based on experimental measurements, was similar to a previous study (Huo and Kassab, 2006). The predictions of the mathematical model showed good agreement with the experimental measurements in control and RVH hearts. A detailed comparison of pulsatile blood flows was made in vessels of various sizes throughout the entire RCA arterial trees between control and RVH.

## Materials and methods

### Anatomical model

Previously, Kassab et al. have carried out morphometric measurements of the RCA-posterior descending arterial (PDA) trees of control and RVH hearts (Kassab et al., 1993). Briefly, the morphometric data on the coronary arterial vessels of diameters <40 μm were obtained from histological specimens and the morphometric data on the coronary arterial vessels of diameters >40 μm were obtained from cast studies. The entire RCA-PDA tree down to the first capillary bifurcations was reconstructed in control and RVH using a growth algorithm (Mittal et al., 2005), based on the experimental measurements of the RV branches excluding the distal tree to the PDA. In summary, the present anatomical mathematical model has exact data (diameters, lengths and connectivity) for the larger vessels and statistical data for the microvessels different from the previous models (Kaimovitz et al., 2005, 2010), which were based on the statistical data (tables with means and standard deviations for diameters, lengths, connectivity) for the entire tree (Kassab et al., 1993).

### Flow simulation

After the branching pattern and vascular geometry of RV branches were generated, a pulsatile flow analysis was performed similar to a previous study (Huo and Kassab, 2006, 2007). Briefly, in the frequency domain, the governing equations (transformed from the conversion of mass and momentum) for flow (*Q*) and pressure (*P*) in a vessel are written as:

(1) $$\begin{array}{r} Q\left(x,\omega \right)=a\cos\left(\omega x/c\right)+b\sin\left(\omega x/c\right) \end{array}$$

(2) $$\begin{array}{r} P\left(x,\omega \right)=iZ_{1}\left[-a\sin\left(\omega x/c\right)+b\cos\left(\omega x/c\right)\right] \end{array}$$

Where *a* and *b* are arbitrary constants of integration, *x* the axial coordinate along the vessel, ω the angular frequency, $c=\sqrt{1-F_{10}\left(\alpha \right)}\cdot c_{0}$ ($c_{0}=\sqrt{\frac{Eh}{\rho R}}$) is the wave velocity, *h*/*R* the ratio of wall thickness to radius, *E* the Young's modulus, ρ the density, and *F*_10_(α)=$\frac{2J_{1}\left(i^{3/2}\alpha \right)}{i^{3/2}\alpha J_{0}\left(i^{3/2}\alpha \right)}$ ($\alpha =\frac{D}{2}\sqrt{\frac{\omega \rho }{\mu }}$, μ is the dynamic viscosity, *J*_0_ the Bessel function of zero order and first kind, and *J*_1_ the Bessel function of first order and first kind). $Y_{0}=\frac{A\left(n\right)}{\rho c_{0}}$(*A(n)* is the cross-sectional area in a vessel) is defined as the characteristic admittance, *Z*_0_ = 1/*Y*_0_ the characteristic impedance, $Y_{1}=Y_{0}\sqrt{1-F_{10}\left(\alpha \right)}$, and $Z_{1}=Z_{0}/\sqrt{1-F_{10}\left(\alpha \right)}$. The impedance and admittance in a vessel is:

(3) $$\begin{array}{rcl} Z\left(x,\omega \right) & = & \frac{P\left(x,\omega \right)}{Q\left(x,\omega \right)}=\frac{iZ_{1}\left[-a\sin\left(\omega x/c\right)\;+\;b\cos\left(\omega x/c\right)\right]}{a\cos\left(\omega x/c\right)\;+\;b\sin\left(\omega x/c\right)} \end{array}$$

(4) $$\begin{array}{rcl} Y\left(x,\omega \right) & = & \frac{1}{Z\left(x,\omega \right)} \end{array}$$

In a given vessel segment, at *x* = 0 and *x* = L, we have the following inlet and outlet impedance:

(5) $$\begin{array}{rcl} Z\left(0,\omega \right) & = & \frac{iZ_{1}b}{a} \end{array}$$

(6) $$\begin{array}{rcl} Z\left(L,\omega \right) & = & \frac{iZ_{1}\left[-a\sin\left(\omega L/c\right)\;+\;b\cos\left(\omega L/c\right)\right]}{a\cos\left(\omega L/c\right)\;+\;b\sin\left(\omega L/c\right)} \end{array}$$

A combination of Equations (5) and (6) yields:

(7) $$\begin{array}{r} Z\left(0,\omega \right)=\frac{iZ_{1}\sin\left(\omega L/c\right)\;+\;Z\left(L,\omega \right)\cos\left(\omega L/c\right)}{\cos\left(\omega L/c\right)\;+\;iY_{1}Z\left(L,\omega \right)\sin\left(\omega L/c\right)} \end{array}$$

Since there are two or more vessels that emanate from the junction points of the entire RCA tree, the junction boundary condition (determined from the continuous pressure and mass conservation at the junction) is written as:

(8) $$\begin{array}{r} Y\left(L\left(mother\right),\omega \right)=\sum Y\left[0\left(daughters\right),\omega \right] \end{array}$$

Equations (7) and (8) were used to calculate the impedance/admittance in the entire coronary tree from inlet to the capillary vessels.

The terminal impedance/admittance of the first capillary was assumed to be equal to the steady value as $\frac{128\mu_{capillary}L_{capillary}}{\left(\pi D_{capillary}^{4}\right)}$ (g·sec/cm^4^), from which we proceeded backwards to iteratively calculate the impedance/admittance in the entire RCA tree of control and RVH hearts using Equations (7) and (8). The pulsatile pressure was the same as the previous study (Huo and Kassab, 2006) and discretized by a Fourier transformation as the inlet boundary condition. The flow and pressure in each vessel were then calculated by using Equations (1) and (2) coupled with the continuous pressure at junctions. The blood flow density (ρ) was assumed to be 1.06 g/cm^3^. The variation of viscosity with vessel diameter and hematocrit was based on Pries' viscosity model (Pries et al., 1992). The coronary wall thickness for every order was adopted from the previous measurements (Guo and Kassab, 2004; Choy and Kassab, 2009). The *in situ* static Young's modulus in control was made as ~8.0 × 10^6^ (dynes/cm^2^) based on the experimental data (Kassab and Molloi, 2001), which was doubled in RVH (Garcia and Kassab, 2009). The dynamic Young's modulus was also considered for various frequencies (Bergel, 1961; Douglas and Greenfield, 1970; Gow et al., 1974), i.e., the Young's modulus increases with the increase of frequency ω (see Figure 2 in Bergel, 1961). Once the flow wave was determined in each vessel, the WSS waves, τ, was calculated as Zheng et al. (2010):

(9) $$\begin{array}{r} \tau \left(x,t\right)=\text{REAL}\left(\frac{4\mu }{\pi R^{3}}Q\left(x,0\right)-\overset{\infty }{\underset{\omega =1}{\sum }}\frac{\frac{\mu Q\left(x,\omega \right)}{\pi R^{3}}\cdot \frac{\Lambda J_{1}\left(\Lambda \right)}{J_{0}\left(\Lambda \right)}}{1-\frac{2J_{1}\left(\Lambda \right)}{\Lambda J_{0}\left(\Lambda \right)}}e^{i\omega t}\right) \end{array}$$

where Λ^2^ = *i*^3^α^2^. Unless otherwise stated, all computations used the previously measured physical properties and parameters as described above.

### Statistical analysis

ANOVA (SigmaStat 3.5) was used to detect statistical differences between control and RVH. A *p* < 0.05 was indicative of a significant difference between the two populations.

## Results

**Figure 1:**
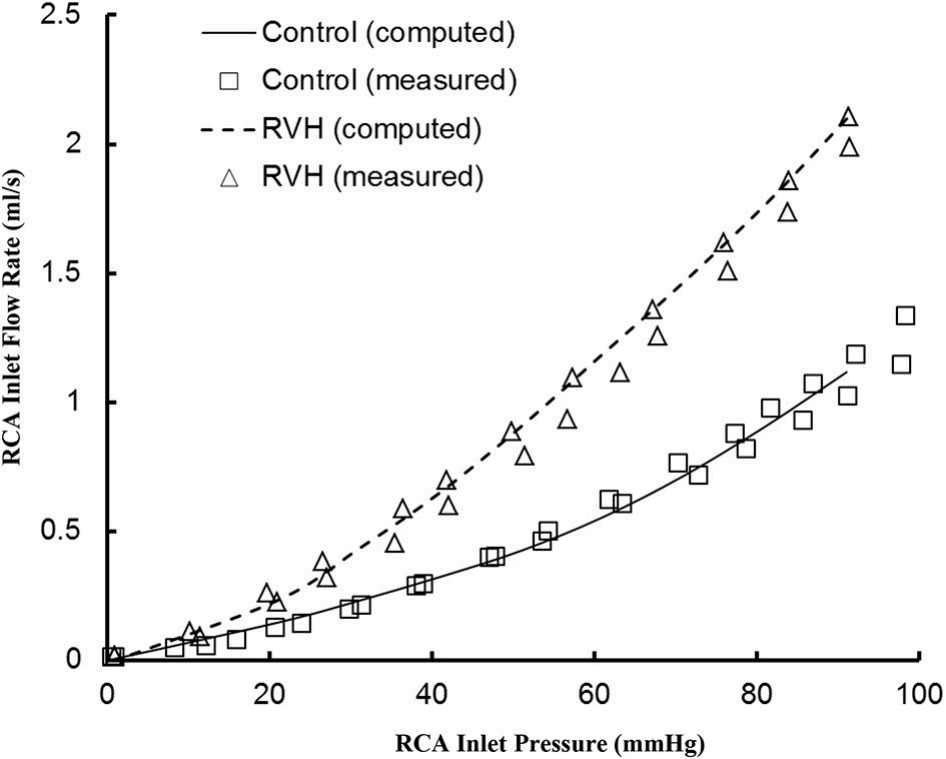
Experimental and computed pressure-flow relationship of the RCA of control and RVH hearts. The experimental results were measured under loading and unloading of pressures as the RCA tree was perfused by cardioplegic solution (μ = 1.1 cp and ρ = 1 g/cm^3^). The pulsatile model was used to compute the pressure and flow at very low frequency (ω → 0).

The pulsatile model has been previously validated experimentally in normal hearts. Here, good agreement was found between experimental measurements and computational results for the RCA of RVH hearts, as shown in Figure 1. The steady-state flows were measured under loading and unloading of pressures as the RCA tree was perfused by cardioplegic solution, which were consistent with the computed pulsatile flows with the frequency approaching to zero since a steady-state flow can be mimicked by a pulsatile flow as ω → 0.

**Figure 2:**
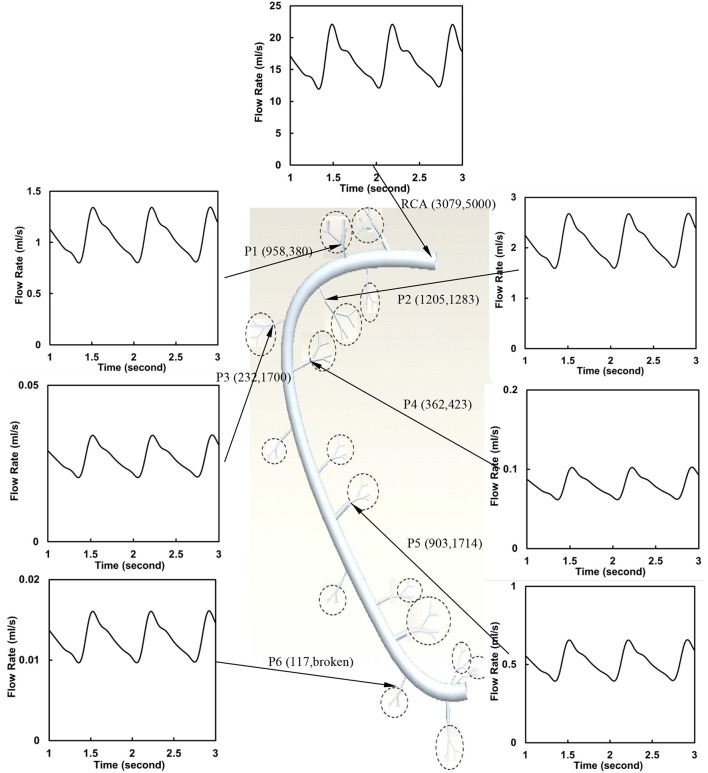
Flow waves at the inlet and primary branches (P1–P6) of RCA tree of a control heart. The dimensions in the parenthesis refer to diameter and length (μm).

**Figure 3:**
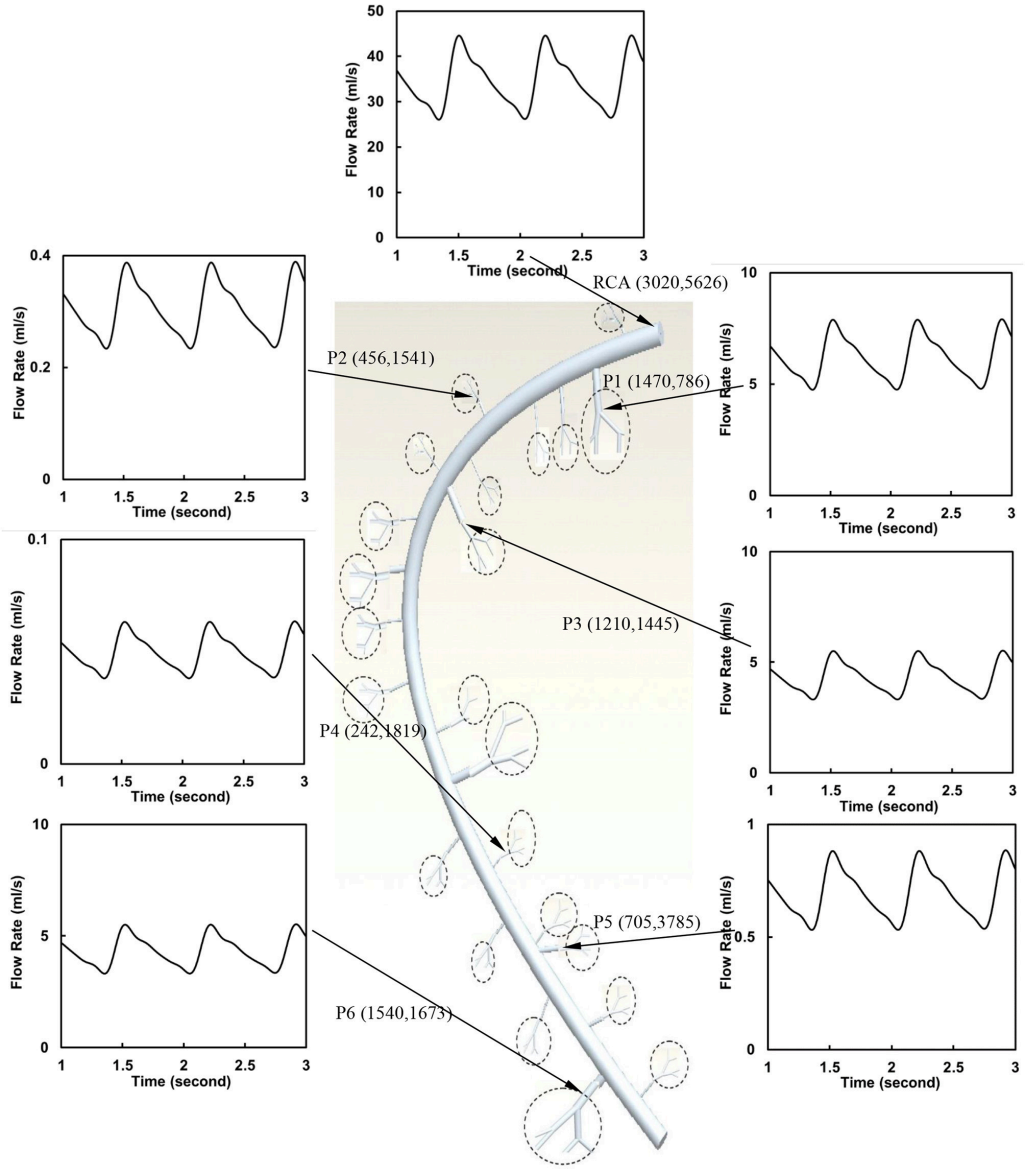
Flow waves at the inlet and primary branches (P1–P6) of RCA tree of a RVH heart. The dimensions in the parenthesis refer to diameter and length (μm).

**Figure 4:**
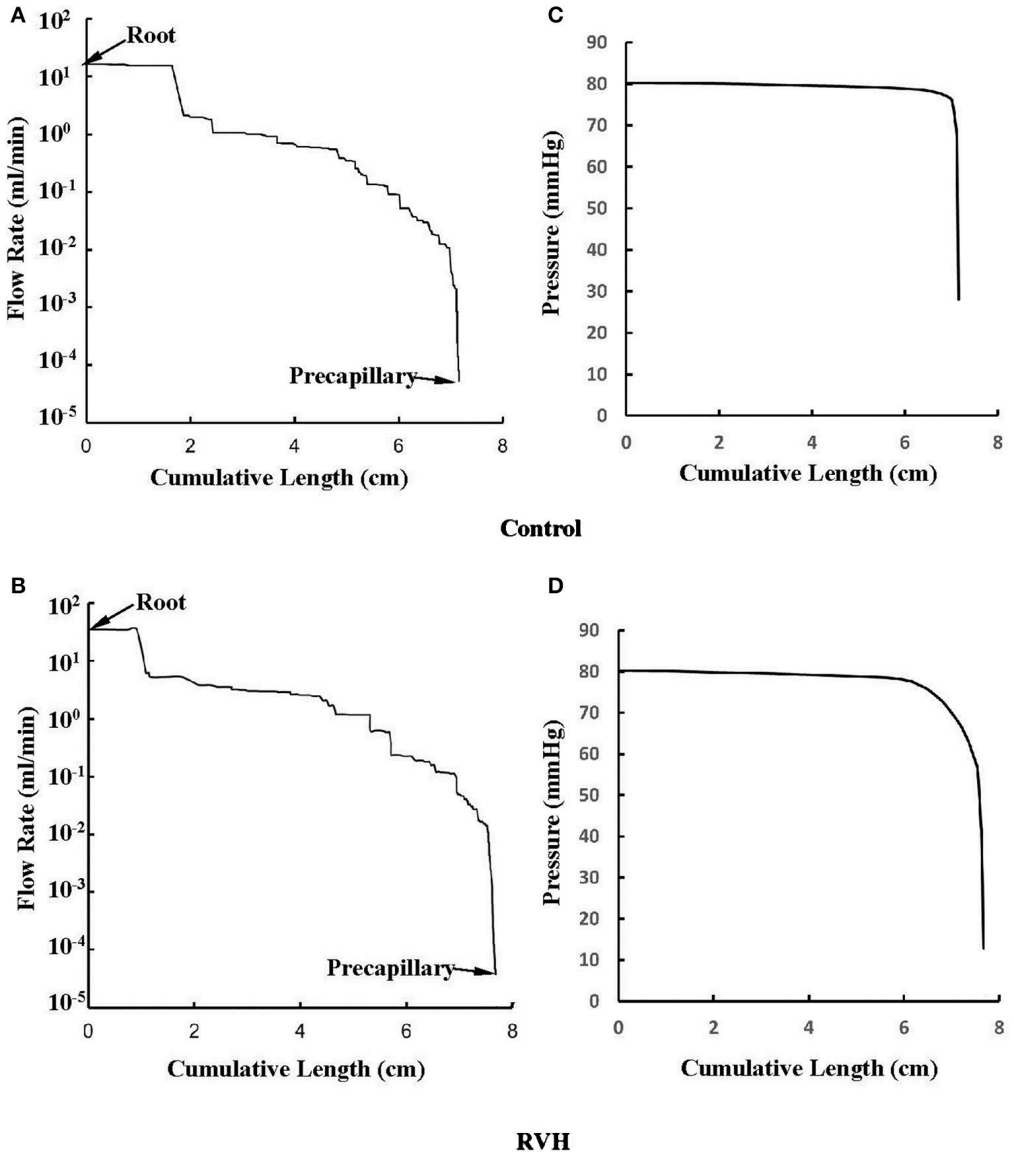
**(A,B)** Relationship between the mean flow (averaged over a cardiac cycle) in a vessel and the cumulative length of the vessel from the root to the precapillary arteriole through similar primary branches in: **(A)** control and **(B)** RVH pig hearts (i.e., P2 in Figure 2 and P1 in Figure 3, respectively). **(C,D)** Relationship between the mean pressure (averaged over a cardiac cycle) in a vessel and the cumulative length of the vessel corresponding to **(A,B)**.

**Figure 5:**
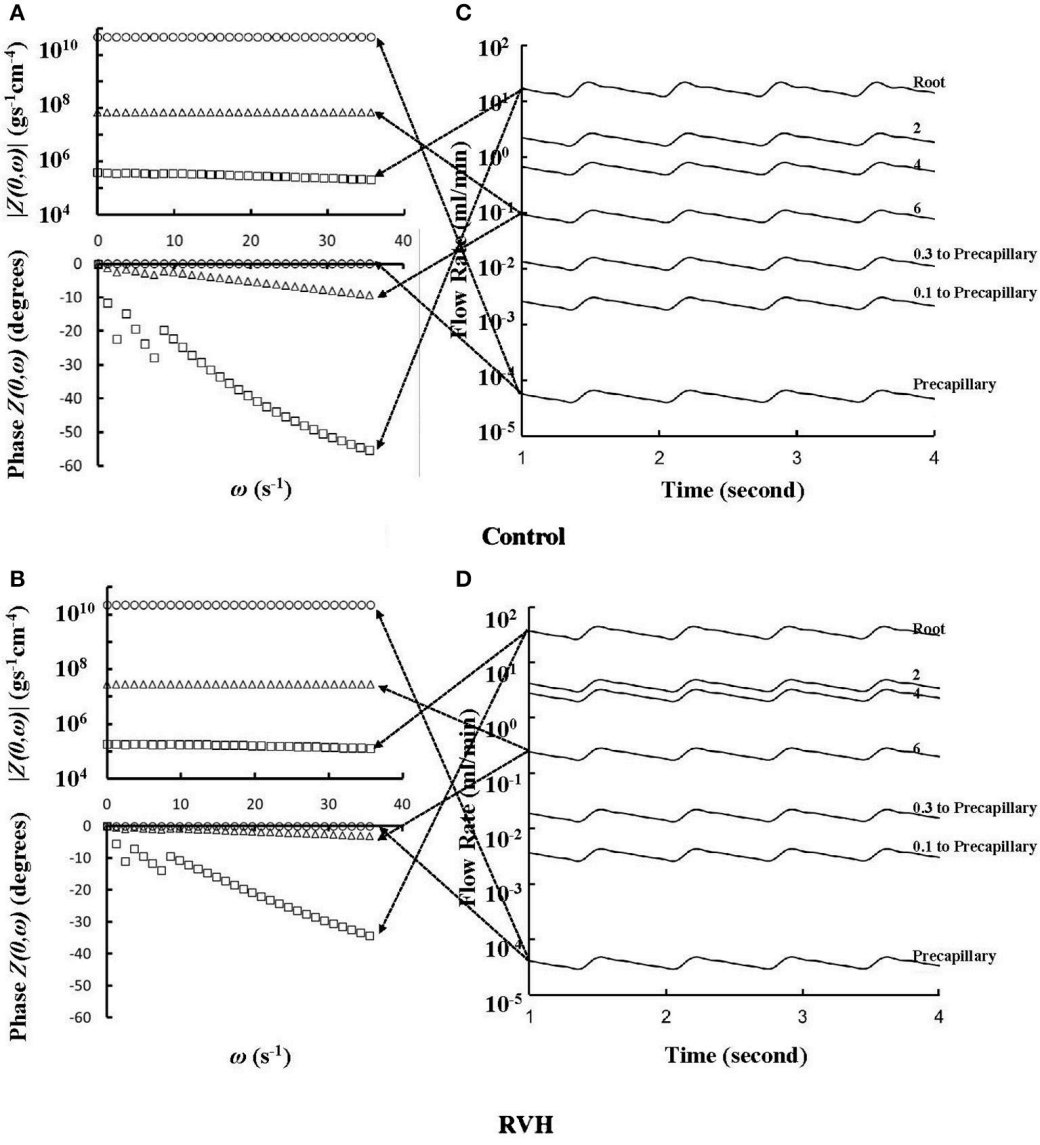
**(A,B)** Impedance |*Z(0*,ω*)*| vs. angular frequency ω and phase *Z(0*,ω*)* vs. angular frequency ω sequentially along the corresponding paths to Figures 4A,B. **(C,D)** Flow waves sequentially along the corresponding paths to Figures 4A,B. The arrows indicate the one-to-one correspondence between **(A–D)**.

The flow waves were calculated in each vessel of RCA tree (excluding the distal tree to the PDA), which has RV branches with a mean (averaged over five anatomic reconstructions) of 0.36 and 1.3 million vessels for control and RVH, respectively. Table 1 summarizes morphometric and hemodynamic parameters in diameter-defined Strahler orders from precapillary arterioles (order 1) to the epicardial RCA tree (the highest order) in control and RVH hearts. The flow waves at the inlet and primary branches of RCA tree of control heart were compared with those in the RVH heart (Figure 2 vs. Figure 3). Given such an increase of vessel numbers, the mean (i.e., time-averaged value over a cardiac cycle) and amplitude (i.e., the change between peak and trough) of flow wave at the inlet of RCA tree in RVH is much larger than that in control. Figures 4A,B show the relationship between the time-averaged flow in a vessel and the cumulative length of the vessel from the RCA to the precapillary arteriole through similar primary branches in control and RVH hearts, respectively. Accordingly, Figures 4C,D show the relationship between the time-averaged pressure in a vessel and the cumulative length of the vessel. Figures 5A,B show the amplitude and phase angle of the impedance in the most proximal RCA, a distal vessel (6 cm from the RCA), and the most distal precapillary arteriole in control and RVH hearts, respectively. Accordingly, Figures 5C,D show a decrease of flow waves sequentially along the path from the RCA to the precapillary arteriole.

**Table 1:** Morphometric and hemodynamic parameters in orders according to the diameter-defined Strahler system.

**Order**	***N***	**Diameter (μm)**	**Flow rate (ml/min)**	**Pressure (mmHg)**
**CONTROL**				
1	93,616	8.77	7.02 × 10^−5^	23.69
2	48,415	11.2	1.61 × 10^−4^	34.59
3	23,302	16.1	4.41 × 10^−4^	45.55
4	8,062	25.8	1.39 × 10^−3^	55.73
5	2,974	47.7	4.69 × 10^−3^	64.32
6	1,010	98.1	1.64 × 10^−2^	74.15
7	400	217	5.40 × 10^−2^	78.38
8	88	491	0.19	79.43
9	70	830	0.63	79.73
10	20	2,420	12.9	79.99
**RVH**				
1	366,758	8.86	7.01 × 10^−5^	22.86
2	190,519	11.7	1.67 × 10^−4^	32.39
3	84,627	16.5	4.62 × 10^−4^	42.37
4	32,275	25.8	1.44 × 10^−3^	52.08
5	8,967	46.8	4.54 × 10^−3^	63.99
6	3,476	91.2	1.54 × 10^−2^	70.32
7	1,255	168	4.98 × 10^−2^	74.28
8	582	314	0.16	76.06
9	205	604	0.58	77.35
10	61	1,241	2.86	79.58
11	25	2,949	22.7	79.94

“*Diameter,” “Flow rate,” and “Pressure” refer to the time-averaged value over a cardiac cycle*.

**Figure 6:**
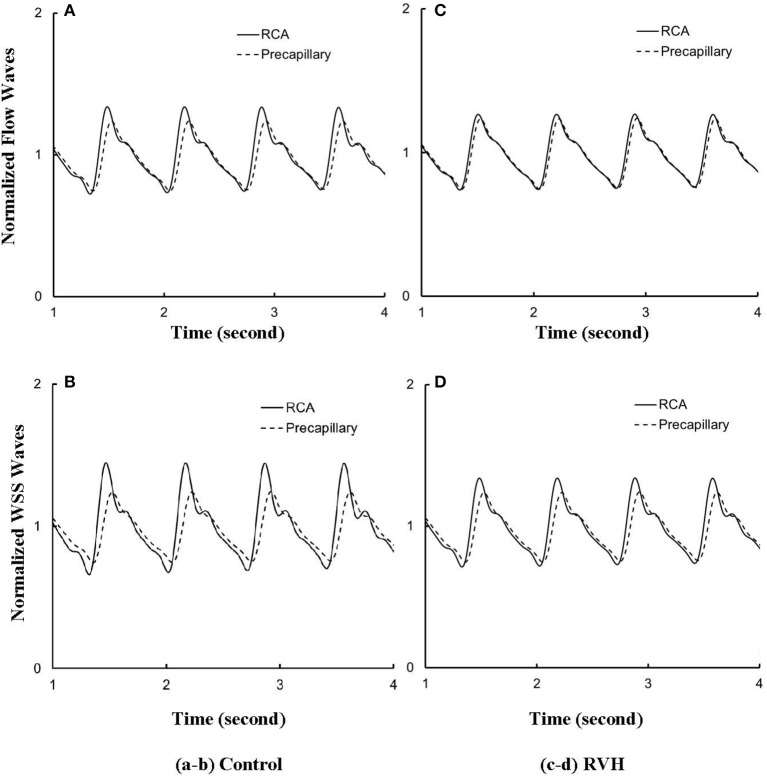
**(A)** Flow and **(B)** WSS waves normalized by the mean values (averaged over a cardiac cycle) at the RCA and precapillary arteriole of control heart. **(C,D)** Normalized flow and WSS waves at the RCA and precapillary arteriole of RVH heart in correspondence with **(A,B)**, respectively.

Despite the large changes of flow waves from the root to the precapillary, we previously reported similar pattern of normalized waves in control hearts. Figures 6A,B show the flow and WSS waves normalized by the mean values at the most proximal RCA and the most distal precapillary arteriole of RCA tree in control. Figures 6C,D show the normalized flow and WSS waves in RVH as compared with those in control. The normalized waves at the RCA and precapillary arteriole are similar to each other (*p* >> 0.05) in both control and RVH. In comparison with the control, the difference of phase angles for flow waves between RCA and precapillary arteriole decreased by about 50% in various frequencies and the pulse wave velocity (PWV) increased by about 40% in the hypertrophic RCA tree.

**Figure 7:**
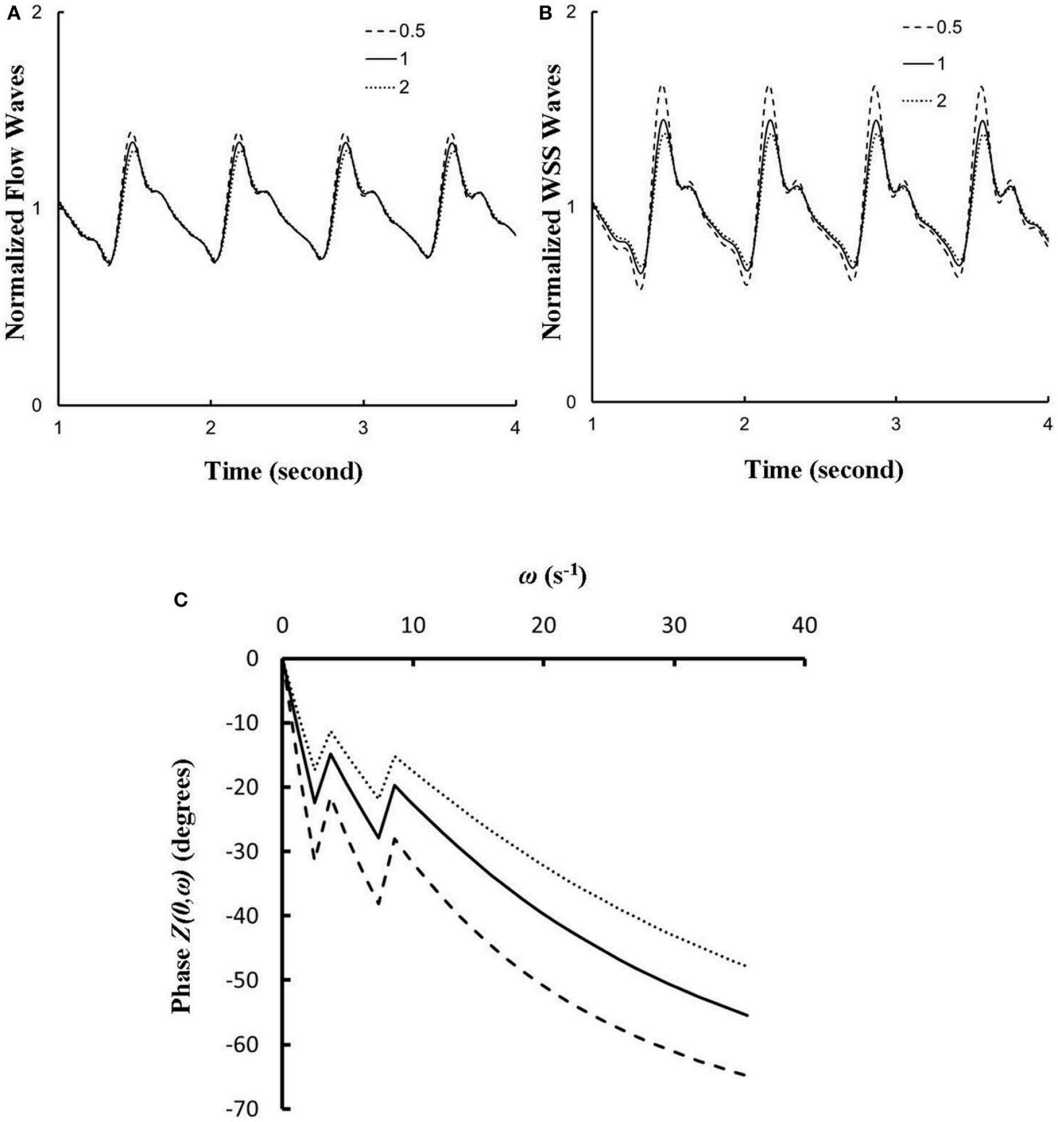
**(A,B)** A sensitivity analysis of normalized **(A**) flow and **(B)** WSS waves at the inlet of RCA tree with a 50% increase/decrease of Young's modulus of each vessel wall, where the baseline (i.e., the unity) refers to the static Young's modulus of 8 × 10^6^ dynes/cm^2^ with adjustment for frequency. **(C)** Phase *Z(0*,ω*)* vs. angular frequency ω at the inlet of RCA tree with a 50% increase/decrease of Young's modulus of each vessel wall.

The effects of vessel compliance on the pattern of flow and WSS waves were examined. Figures 7A,B show a sensitivity analysis of normalized flow and WSS waves, respectively, at the inlet of RCA tree of control hearts with a 50% increase/decrease of Young's modulus of each vessel wall (including both static and dynamic Young's moduli). Figure 7C shows the change of the phase angle of the inlet impedance with a 50% increase/decrease of Young's modulus of each vessel wall. The change of vessel compliance in physiological range had negligible effects on the normalized waves (*p* >> 0.05), but significantly affected the phase angle of the impedance.

## Discussion

The major finding was that RVH maintained similar patterns of normalized flow and WSS waves in different size vessels. RVH, however, did significant increase in the amplitude and mean (time-averaged over a cardiac cycle) of waves in large epicardial coronary branches due to the increase in number of small arterioles.

### Steady-state flow vs. pulsatile flow

Based on morphometric measurements and steady-state hemodynamic analysis, the remodeling of structure and function of the entire RCA tree was determined in RVH after 5 weeks of pulmonary banding (Kassab et al., 1993; Huo et al., 2007). The number of vessel segments in RVH was about four times larger than that in control hearts. The steady-state flow (which approximately equaled to the time-averaged value of pulsatile flow) was significantly increased in the large epicardial vessels in RVH, but distributed through substantially more vessels such that the flow and WSS in the small perfusion arterioles were not statistically different from the control (Huo et al., 2007). Here, we compared pulsatile flows between control and RVH using the Womersley-type model (Huo and Kassab, 2006) and morphometric measurements (Kassab et al., 1993; Kassab and Molloi, 2001; Garcia and Kassab, 2009). Corresponding to the steady-state flow, the amplitudes of flow and WSS waves in RVH were significantly increased in the epicardial vessels, but similar to those in the perfusion arterioles of control, as shown in Table 1 and Figures 2, 3. The amplitude and mean of flow and pressure waves decreased along the path from the root down to the precapillary arteriole in the RVH, which has the same trend as the control (see Figure 4) and also agrees with a previous study (Huo and Kassab, 2006). The increase of stiffness in RVH reduces the phase angle of vessel impedance (see Figure 5) and leads to a significant decrease (~50%) of phase angle difference for flow waves between the most proximal RCA and the most distal precapillary arteriole in various frequencies (see Figure 6), which reflects an about 40% increase of PWV in the RCA tree of RVH. Although an increase of aortic PWV is widely known as a marker of cardiovascular risk in hypertension (Mohiaddin et al., 1993; Butlin et al., 2013), it still requires further investigation in the interaction of large and small arteries, given the unknown causal relation between stiffening and hypertension in vessels of various sizes.

### Flow and WSS waves

The flow waves at various coronary vessels of diastolic control hearts were found to have a tendency of scaling to a single curve, except for a small phrase angle difference, when they were normalized by the mean values (Huo and Kassab, 2007). In the beating hearts, Ashikawa et al. measured the flow velocities in small arterioles (diameter of 12.8 ± 4.1 μm), capillaries, and small venules (diameter of 16.5 ± 6.5 μm; Ashikawa et al., 1986) and Toyota et al. measured the flow velocities in arterioles (diameter of ~100 μm; Toyota et al., 2005), which also showed similar normalized flow waves. Here, the normalized flow and WSS waves in the RCA tree of RVH maintained the same scaling characteristic, as shown in Figures 5, 6. Moreover, the sensitivity analysis in Figures 7A,B showed the change of stiffness of RCA tree in physiological range, which had negligible effect on the normalized flow and WSS waves. This can be explained from the constitutive equation [i.e., $\frac{A}{A_{ref}}=\frac{\left(p-p_{ref}\right)\cdot \left(R/h\right)}{E}+1$, in which *A* is the CSA, *p* the pressure, *h*/*R* the ratio of wall thickness to radius, *E* the Young's modulus]. From the equation, Young's modulus (the denominator) is almost twenty times larger than (*p*−*p*_*ref*_)·(*R*/*h*) (the numerator) in normal coronary arterial tree such that $\frac{\left(p-p_{ref}\right)\cdot \left(R/h\right)}{E}$ is much less than one, where the static Young's modulus in control is 8 × 10^6^ dynes/cm^2^ and increases as frequency increases, (*p*−*p*_*ref*_) varies from −20 to +20 mmHg, and (*R*/*h*) approximately equals to ten. A 50% perturbation for the stiffness had negligible effect on the waves.

### Physiological implications

Coronary blood vessel wall is subjected to various types of hemodynamic forces (e.g., hydrostatic pressure, cyclic stretch, and fluid shear stress) caused by the pulsatile blood pressure and flow (Chiu and Chien, 2011). Intraspecific scaling power laws of vascular trees, derived from the steady-state analysis (Huo and Kassab, 2012), characterize coronary vasculature with remarkable simplicity. The compensatory remodeling in RVH was found to maintain the structure-function hierarchy (preserved scaling exponents) of fractal-like coronary arterial tree (Gong et al., 2016). Furthermore, the present study shows the preserved shape of normalized wave of pulsatile blood flows in the entire coronary arterial tree, which is unchanged during the remodeling in RVH. This implies that intraspecific scaling power laws of vascular trees can be extended from the steady to dynamic states. On the other hand, the altered phase angle of vessel impedance and flow and WSS waves shows the effects of the remodeling on the pulsatile blood pressure and flow in RVH, which requires further investigations.

### Critique of study

The present study carried out the pulsatile flow analysis based on the morphometric data and physiological measurements, but the constitutive equation was determined in a vasodilated state in the absence of vasomotor tone where coronary flow reserve was substantially reduced. The stiffness of large arteries was assumed to equal to that of small arterioles. Future studies are needed to identify the contribution of vascular tone to the vascular remodeling and flow waves in RVH with consideration of the stiffness in various size vessels.

### Significance of study

RVH is a compensatory response to pulmonary hypertension and the compensatory adaptations of coronary circulation serve to maintain perfusion of the increased myocardial mass. Here, we showed that the shape of normalized flow and WSS waves was preserved despite a significant increase of the amplitude and mean of flow and WSS waves in the epicardial vessels. The increase of stiffness in RVH increased the PWV in coronary arteries and reduced the phase angle difference for flow waves between the most proximal RCA and the most distal precapillary arteriole, but had negligible effect on the pattern of flow and WSS waves. The precise prediction of flow and WSS waves in coronary microcirculation is physiologically and clinically important to understand the ventricular hypertrophy.

## Author contributions

Data analysis were performed by YH at the college of engineering, Peking University. Paper was drafted and revised by YH and GK at the California Medical Innovations Institute.

### Conflict of interest statement

The authors declare that the research was conducted in the absence of any commercial or financial relationships that could be construed as a potential conflict of interest.
